# Exploring experiences and needs of spousal carers of people with behavioural variant frontotemporal dementia (bvFTD) including those with familial FTD (fFTD): a qualitative study

**DOI:** 10.1186/s12877-022-02867-1

**Published:** 2022-03-07

**Authors:** Sara Tookey, Caroline V. Greaves, Jonathan D. Rohrer, Roopal Desai, Joshua Stott

**Affiliations:** 1 ADAPT Lab, Research Department of Clinical, Educational and Health Psychology, University College London, 1-19 Torrington Place, London, WC1E 7HB UK; 2 Dementia Research Centre, Department of Neurodegenerative Disease, UCL Queen Square Institute of Neurology, London, UK

## Abstract

**Introduction:**

Carers of people with frontotemporal dementia (FTD) experience greater challenges than carers of people with other dementias due to the younger age of onset and the challenging presentation of symptoms. The aim of the present study was to explore experiences of spousal carers of people with bvFTD, including those with the familial form of the disease (fFTD).

**Method:**

Fourteen qualitative interviews were analysed using an inductive approach to Thematic Analysis to understand experiences of spousal carers of people with bvFTD including those with fFTD.

**Results:**

Five main themes were identified including: a) The “Constant Battle” – A journey toward an FTD diagnosis, b) Shock, Relief and Fear – Challenges persist post diagnosis, c) The “Life Altering” impact – The loss of the spousal relationship and shifting roles, d) Adapting, Managing Symptoms and Receiving Carer Support, e) Lack of General Knowledge – Barriers to support.

**Conclusions:**

Healthcare professionals should be educated on the initial presentations of FTD, to enable carers and families receive timely diagnosis and appropriate support. Future research should investigate the impact of fFTD on carers and families, to explore positive or meaningful experiences in caring, as well as theory-driven research to identify helpful coping strategies for carers of people with FTD.

**Supplementary Information:**

The online version contains supplementary material available at 10.1186/s12877-022-02867-1.

## Introduction

Frontotemporal dementia (FTD) is the second most common cause of dementia in people under the age of 65 years [1], and is attributed to 25% of individuals with dementia in those over 65 [2]. FTD is a neurodegenerative condition that impairs executive functioning and language, impacting on behaviour, planning, problem-solving, emotional control and speech [3]. FTD encompasses a behavioural variant (bvFTD) and a language variant, known as primary progressive aphasia (PPA) [4, 5]. The language variant can be further subdivided into semantic (svPPA), non-fluent (nfvPPA) and logopenic variants (lvPPA), however lvPPA is often associated with underlying Alzheimer’s disease pathology [4, 5].

Cases with a family history (~ 30–40% of cases) are referred to as familial FTD (fFTD) [6]. The majority of fFTD is caused by a genetic mutation inherited in an autosomal dominant manner, usually in one of three genes, *C9orf72, MAPT* and *GRN* [6], meaning that a child born to a parent with the disease has a 50% chance of inheriting the mutation [7]. fFTD can present in all of the variants, but most people develop bvFTD, which is the focus of this study [6].

New diagnostic criteria for bvFTD were developed in 2011, when the International Behavioural Variant FTD Criteria Consortium (FTDC) used pathologically confirmed cases of FTD to create sensitive and specific clinical criteria for diagnosing bvFTD [8]. The criteria identified six behavioural and cognitive symptoms central to the diagnosis: i) apathy, ii) behavioural disinhibition, iii) loss of sympathy or empathy, iv) perseverative or compulsive behaviour, v) hyperorality and dietary changes, and vi) executive and language dysfunction. BvFTD diagnosis requires three of these symptoms to be present [9]. Despite these criteria, symptoms are often misdiagnosed by healthcare professionals as a psychiatric illness before reaching a diagnosis of bvFTD [1, 3, 10].

Carers of people with FTD (whether bvFTD or FTD in general) experience greater overall burden [11–14], stress and depression [15] than carers of people with typical, late-onset forms of dementia such as Alzheimer’s disease. This is probably due in part to younger age of onset and the genetic link [3], but it is the behavioural changes in FTD that are often reported as the most stressful by carers [11, 16–18]. As a consequence of the disease, carers describe a loss of the interpersonal relationship with the person who was once seen as a companion, spouse, lover and friend [19–21]. In the UK support for caregivers of people with dementia is largely provided within the community through primary care (general practitioners) and specialist memory services. As such much of this is designed for AD and older adults. Support for those with rare and early onset dementias is much less common. There is, therefore, significant geographical variation in services available to individuals within their community.

There is a breadth of literature exploring the needs of carers of people with typical forms of dementia, such as Alzheimer’s disease and vascular dementia. Interventions for these groups have shown promise in improving carer wellbeing and reducing burden associated with caring. However, it is unclear if these interventions (that have been developed to support carers of people living with dementia in general) are effective for carers of bvFTD. Furthermore, there are few studies exploring the subjective experiences and needs of family carers of people with bvFTD [22] and a small number of interventions piloted in caregivers of those with FTD, or both FTD and Alzheimer’s disease together [23–25]. To our knowledge, there have been no randomised control trials and there are no specific interventions developed to support caregivers of people with behavioural variant FTD. This is particularly so in the area of fFTD where there may be implications for young families coping with the genetic version of the disease. It is important to consider the specific issues experienced by those caring for people with FTD as general dementia support services available within the community are often not appropriate. For example, the earlier age at onset often experienced in FTD can cause increased strain on caregivers who are often still in employment and may also have a family to take care of. Additionally, for those with fFTD, there is often the added issue of heritability, as caregivers also take on the burden of their children living at-risk of FTD. Despite an increase in research studies in the past decade to understand FTD pathology, genetic links, and prognosis, literature on understanding the psychosocial needs of families of people living with this debilitating disease is limited [26]. Few studies have employed a qualitative methodology, and the studies that are available have used very small sample sizes [19], and mixed multiple variants of FTD together when reporting their findings [27]. These are limitations of the existing literature, and raises concerns regarding the transferability of its findings to carers of people with bvFTD and fFTD.

The current research aims to identify the psychosocial support needs and experiences of spousal carers of people with bvFTD and fFTD. The analyses will identify barriers and facilitators of getting these needs met, and will highlight potential similarities and differences across a group of non-genetic (sporadic) bvFTD and a group with a genetic form of this subtype (bvFTD due to fFTD).

## Method

The current study took a phenomenological stance to understand the experiences of carers of people with bvFTD and fFTD, and explored their views, perceptions and experiences of being a spousal carer of a person with bvFTD, as well as the meaning they attach to these.

### Participants and setting

Participants were 14 spousal carers living with a person diagnosed with bvFTD, five of whom were caring for people with sporadic FTD, and nine of whom were caring for people with fFTD. Inclusion criteria were: a) membership of an FTD support group, b) a main (Spousal) carer of a person with bvFTD. Carers with a diagnosis of a major psychiatric disorder, learning disability, or individuals unable to understand English, were excluded. Interviews were conducted three months to 11 years post diagnosis, with nine being diagnosed within one year of participating in the study. Characteristics of carers and care recipients are reported in Table 1.

### Recruitment and data collection

Participants were identified from two existing UCL Queen Square Institute of Neurology Dementia Research Centre studies, which recruited participants from across the United Kingdom; the Longitudinal Investigation of FTD (LIFTD), and the GENetic Frontotemporal dementia Initiative (GENFI). The sample was initially recruited to have an even split between carers of people with sporadic and familial bvFTD. However, a more opportunistic sampling was later used to reach thematic saturation, with a sample of 14 participants recruited over a period of seven months. Participants were provided with information regarding the interview study by CG either verbally or via email. Interviews were conducted in person (*n* = 5), via Skype (*n* = 8) or telephone (*n* = 1), depending on participant preference. Written informed consent and ethical approval was obtained. Participant confidentiality was maintained by assigning pseudonyms.

In-depth, semi-structured interviews were conducted in order to explore carers’ support needs and experiences of being carers of people with bvFTD and fFTD. Development of the interview schedule was informed by a literature review [24] and developed in collaboration with the LIFTD and GENFI research teams and piloting with a bvFTD carer. The expert by experience pilot interview was not included in the final sample. The interview explored the impact of FTD on participants’ lives, their positive and negative experiences, and explored their support needs (see Appendix A for the interview schedule). Interviews were conducted by an experienced qualitative researcher (ST), ranged in length between 45–90 min and took place between April and November 2019. All interviews were audio-recorded and transcribed verbatim. The researcher (ST) explored and attended to preconceptions and biases prior to the analysis with the aim of avoiding interference with data collection and interpretation.

## Analysis

Interviews were analysed using an inductive and reflexive approach to Thematic Analysis [28]. Patterns and themes across a dataset and the differences between sporadic and familial bvFTD were also the focus of the analysis.

A systematic six-step process was followed according to the steps outlined for this method [29, 30] and NVivo software (Version 12, for Mac) was used to aid in the process of coding and analysis. Analysis steps included: i) data familiarisation ii) initial coding iii) searching for themes by grouping codes iv) checking the themes v) defining and naming the themes vi) report writing. Initial development of themes was reviewed by the senior author. Thirteen participants were approached to review the write-up of the results, two participants responded and no changes were required as a result of this process. The remaining 14^th^ participant was not approached for review due to health issues and unavailability by email.

## Results

Five main themes were identified to depict key aspects of understanding the experiences and needs of spousal carers of people with FTD. Main themes included: (i) *The “Constant Battle” – A journey toward an FTD diagnosis*, (ii) *Shock, Relief and Fear – Challenges persist post diagnosis,* (iii) *The “Life Altering” impact – The loss of the spousal relationship and shifting roles*, (iv) *Adapting, managing symptoms and receiving support*, (v) *Lack of general knowledge- Barriers to support for spousal carers of FTD.* See Table 2 for the representation of themes across participants.

### The “Constant Battle” – A journey toward an FTD diagnosis

All of the carers expressed having been through a challenging road to receiving a diagnosis for their spouses’ bvFTD. Challenging stages along this journey included: noticing slow and subtle symptoms, misattributing symptoms to situational, psychological or relationship problems, and feeling isolated in the experience leading up to diagnosis. The excerpt below demonstrates the experience of noticing the initial symptoms of FTD as subtle and gradual changes in the personality and behaviour of her spouse.

*C7: It was so gradual. I mean, we're talking sort of over a year, if you're with him all the time. You wouldn't have noticed it[...] But it was it was so subtle, and it wasn't too odd..* (Female, fFTD)

Another carer outlined the process of trying to make sense of the initial changes she was observing with her husband’s behaviour and attributing symptoms of aggression, impulsivity and loss of empathy to psychological or relationship difficulties in their marriage.

*C14: Because when he first started behaving differently, I thought it was just a mid-life crisis or our marriage breaking down. So I was on the Internet, looking for ‘mid-life crisis’. When I thought he was just being difficult or awkward, and I'd ask him to do something, he wasn't listening when I was talking to him, and he wasn't taking my worries or concerns or feelings [into account].* (Female, fFTD)

In an attempt to make sense of symptoms carers sought out information that could help them to understand their partner’s unusual and at times distressing behavioural and personality changes. During this process, carers described feeling socially isolated when family, friends or professionals did not take concerns seriously.

*C7: I felt like I was completely on my own in saying, “it's not eccentricity. It's not him getting old. It's not stress”[…] So [before receiving the diagnosis] it was a constant battle with explaining for him what was going on.* (Female, fFTD)

Barriers experienced in medical services included initial misdiagnoses of other physical or psychiatric conditions and various mental health conditions. Carers described feeling as though concerns were not taken seriously by medical professionals, and that they needed to “bully” (C2) or push for further testing to reach an appropriate diagnosis.

*C7: He was diagnosed with bipolar, hypomanic, bipolar or something. […] See I'd Googled, as you do, and I thought he'd got Pick's disease. And I told the people at the memory clinic, "I think it's this." And they said, "It's not. He's passed his memory test."* (Female, fFTD)

C7 then recounted having to further justify seeking a new diagnosis by acquiring information about the condition to inform the dementia services clinic about FTD, and advocate for her spouse to receive further specialist diagnostic testing.

### Shock, relief and fear – challenges persist post diagnosis

Upon receipt of a FTD diagnosis, 12 carers reported experiencing mixed feelings of relief and fear about the future, and a continued sense of isolation, given the lack of support offered after receiving the diagnosis.

*C8: When he got the diagnosis, I wasn't happy, but I was relieved that I knew that there was something wrong with him and it wasn't my imagination. […] I kind of felt a bit naively, "Oh, this will be all right. We'll deal with this. I'll just sit in front of the TV and he'll sit there quietly and we'll get on." But I didn't realize with this disease that he's got, it doesn't work like that. I didn't really know what was going to happen.* (Female, sporadic bvFTD)

After receiving the diagnosis, some carers described feeling a sense of relief that they now had a label that could explain their spouse’s behaviour. Out of the 12 carers who spoke about their experience of receiving a diagnosis, all but one of the carers (C3) expressed having continued difficulty coping with the uncertainty regarding managing expectations of their spouse's future following diagnosis.

*C9: They have termed it, it's genetic and it's progressive. […] Everybody is slightly different. They've all got their own things, but they will get worse. It may become that he gets weaker or he may get violent […] I thought, ‘oh this is going to be dreadful. I'm never going to cope with this.’* (Female, fFTD)

C14 expressed her surprise at the lack of resources received following diagnosis, by contrasting this with receiving a diagnosis of cancer,

*C14: It's [not] like cancer. He had cancer... And when he was diagnosed with that he went in for a follow-up every three months. But with the FTD, he's never seen anybody since. And you are kind of left on your own.* (Female, fFTD)

### The life-altering impact – the loss of the spousal relationship and shifting roles

All 14 participants made reference to the many life-altering aspects of becoming a spousal carer for a person with bvFTD. Participants described having difficulty managing caring duties alongside increasing responsibilities in the household, shifting roles, addressing their family’s future risk, and experiencing a deep sense of loss as a result of no longer feeling able to connect or communicate with their spouse.

*C7: The main challenge- it's- you go from a family unit with two parents and, you know, doing everything together, to a single parent, which is one thing. But then it's a single parent with a parent- because there's somebody there, but you've got responsibilities, plus you've got someone else to look out for.* (Female, fFTD)

Carers additionally described the difficulties of managing responsibilities in the home and the emotional toll that their spouse's behavioural symptoms had on them. Eight carers described difficulties related to sleep as the most significant issue for them. Participants described feeling exhausted and having difficulties falling or staying asleep as a result of their spouse’s inconsistent or interrupted sleeping behaviour. Carers described their own lack of sleep as the main contributor to frustration, lack of patience with their spouse and difficulty coping with caring responsibilities. Others reported that some of the care-recipients slept more than they used to, and this allowed them time for respite in the day, while one carer described oversleeping as an issue that interfered with her ability to share quality time with her spouse (C14).

*C1: I do get angry. It’s lack of sleep really… With no sleep, with disruption in your sleep it’s just very very hard... And I feel now that FTD has stolen my patience in a way, because of the situation.* (Female, fFTD)

One of the experiences described by spousal carers as the most difficult was the experience of loss, described by seven participants, who expressed having difficulty coming to terms with the reality that they could no longer connect or communicate with their spouse or life partner. This created a deep sense of loss (for example of empathy and memory), with carers likening it to losing a life partner along with their previously held hopes and expectations for their future together.

*C3: If you think about it, you’re managing somebody’s life who’s there with you, but you can’t really have any discussion about it what-so-ever, because if you do, it’s forgotten about in seconds and he doesn’t understand the implications. [...] He doesn’t have that emotional response really. So, there’s no point discussing.* (Female, sporadic bvFTD)

Four carers likened these difficulties in communicating and changing roles to that of caring for a child. However, they clarified this by stating that unlike children, their spouse had a diminishing capacity to learn and would become increasingly more dependent on them to meet their needs.

*C3: At first I felt really awful about almost taking over someone’s life and saying what they could and couldn’t do. But then I did it with children.... And I’ve almost seen that I’m doing that just to manage him now, because he can’t make those decisions for himself. But of course he thinks he can, and that’s always the problem..., I have to treat him, in a way, like he’s a difficult child really.* (Female, bvFTD)

Carers of spouses diagnosed with the familial form of bvFTD (fFTD) described the impact of identifying a strong genetic link and the implications this holds for both the carer and the future of young family members. Carers expressed that they felt a deep sense of sadness, grief and fear when thinking about the genetic implications of the disease on their children, and feeling a sense of helplessness in their ability to support their children in the future, as exemplified in the excerpt below.

*C6: I have to be careful with her (my daughter), because she sees her dad and I know at the back of the mind she's looking at him and thinking, 'that could be me'. […] I don't tell her everything that he does, or I don't offload to her; I'm not going to because she's my baby and I want to make her better, and I can't. […] That's kind of hard.* (Female, fFTD)

Other carers of spouses with fFTD described the emotional challenge of coming to the decision to inform their families of the genetic link, trying to decide when their family would be ‘ready’ to receive news about their own risk of developing the disease.

*C1: He (the consultant) thought I should tell my children right away, but I don’t feel it’s the right time at the moment. [...] They’ve got a lot of stress in their lives at the moment, and I don’t feel that’s right. So I’m holding onto that for a moment. [It’s] difficult.* (Female, fFTD)

### Adapting, managing symptoms and receiving carer support

All carers in the study identified the importance of learning to adapt to the caring role, and trying to find ways of managing the challenging symptoms, particularly the behavioural symptoms of their spouse. Carers also expressed the value of receiving support to meet the specific needs of spousal carers of people with bvFTD.

### Appreciating, adapting and living in the present

Eleven carers described the benefits of holding an adaptive and positive mind-set in coping with and adapting to the role of becoming a spousal carer. Carers expressed the usefulness of practising appreciation for the positive aspects of their life that were maintained despite the deteriorating illness of their spouse. Examples of ways that carers practised this positive perspective-taking included comparing their situation to *those who “have it far more severely*” (C10), practising gratitude (C8 below), and implementing transferable skills from other caring roles they had previously occupied. Carers also expressed the helpfulness of planning for the future while also trying to focus on “living day to day” (C12).

*C8: Now I realise that possessions don't mean anything […] You know, nobody, nothing can give you what you want back, which is your partner's health. So there's nothing out there that you need. So you then become just happy in your surroundings with what you've got, and just living that life now and making it the best for them that you can,* (Female, bvFTD).

Carers of people with fFTD expressed *a “bubble of hope”* (C14) that came from participating in a trial treatment, which they believed might halt the deterioration of the illness for their spouse, or could offer hope for future generations.

### Managing challenging behavioural symptoms of FTD

Behavioural symptoms described as challenging included lack of insight, apathy and compulsivity. These changes were described as confusing, sad, and difficult to manage in public or embarrassing at best. At their worst, they placed the person with bvFTD and others at increased risk of harm. Therefore, carers put in place strategies to manage challenging and risky behavioural symptoms. As C10 describes the experience of trying to manage these risks below:

*C10: He was going to hang himself in the woods in the park […] And then [when we came back home] I wondered why he was nearly falling over. I realised it's because he was picking up alcohol and drinking […] And yes, I had to empty our house of alcohol and knives and everything.* (Female, fFTD)

Other carers discussed applying other restrictions to reduce risk, which included withholding money to reduce the risk of being taken advantage of by others, or to reduce risk of compulsive spending (C3) or controlling access to food (C14, C4, C6).

Other carers described a difficult experience of having to manage their spouses’ risky behaviour toward others, including violence and aggression.

*C8: He started to dislike that son and then he started to dislike me. […] And then that was when the violence started. […] And I didn't let him in the house for a week. I just was so confused. I was so upset. […] He'd have this outburst and then a while later it was, we're all calm now […] And he never spoke about it.* (Female, bvFTD)

Another key difficulty for carers was managing their spouse’s behavioural symptoms in public. Carers described needing to put a system of control in place, to monitor, supervise or limit their spouse’s behaviour when in a public space as exemplified by C10 below.

*C10: Here was that feeling of embarrassment for me as well, because he comes out and says odd things. And I don't like that. I'm a very quiet, private person, and things like this I find extremely hard. Yes, that's a bit hard- more than hard to accept.* (Female, fFTD)

### Information gathering and linking to specialist services

All of the carers acknowledged the value of acquiring knowledge about FTD, connecting with available resources to provide information, advice and carer support or respite. Thirteen carers described the value of obtaining information to educate themselves about the specific symptoms of bvFTD to help them manage expectations of disease progression, and provide helpful tips and resources to support them in their caring responsibilities. Carers did this by seeking out opportunities to learn about FTD from online sources, professionals and other carers.

Eleven carers described the helpfulness of speaking with family, friends, other carers and professionals to gain an understanding of what to expect, and to normalise challenges faced in caring for a person with FTD. Others (e.g. C11) discussed that specialist FTD support was “*much more helpful”* than generic support, highlighting the necessity for services to accommodate for the specific needs of carers of people with FTD. These were understood to be different from those caring for people with more general forms of dementia. Six carers identified the importance and usefulness of accessing information and emotional support online.

Three participants expressed mixed feelings about the value of connecting with fellow FTD carers whilst acknowledging that there may be a time in the future when they would find attending carer support groups helpful. Whilst not all carers agreed on the value of using carer support groups, 13 carers identified carer respite as one of the most essential needs amongst spousal carers of people with FTD.

*C1: I realised that being together 24/7 was a nightmare for both of us, and that I needed a break, but he needed a break away from me. […] I decided that I really wanted to go to [dementia charity choir] so I got a carer […] I go back to choir once a week, and a girl comes in to sit with him,*

Other carers described similar experiences of attending social activities run by dementia support charities, and described them as helpful in enabling them to continue to participate in activities and hobbies of interest while receiving support and respite from daily spousal caring. These connective spaces for carers provided an opportunity to spend time with others with a shared understanding of the carer’s experience. For many, this formed an important part of the carer’s social supportive network post-diagnosis.

*C10: I am grateful that I have got a lot of friends and people I knew, and the vast majority coming in now are people we know from the dementia cafes and charity things.* (Female, fFTD).

Outside of informal social support and networking spaces, 10 carers highlighted the importance of attending medical follow-ups or receiving nursing support as a way of providing on-going support after diagnosis and during the care-recipients’ lifespan.

*C8: I've used Admiral nurses* [registered nurses who specialise in dementia]. *I've used those quite a few times and they are lovely. They are so supportive and so understanding, because he's been very, very violent to my neighbours* (Female, sporadic bvFTD).

Five of the nine participants caring for a spouse with fFTD expressed the value of having prior knowledge about dementia from their spouse’s family. This knowledge, prior to diagnosis, was helpful in preparing them to manage expectations.

*C1: We’ve known for quite a long time, because his sister had it…,* (Female, fFTD)

The excerpts above highlight the needs of carers by identifying the ways in which they adapt to the new carer role, manage symptoms, and receive crucial support from healthcare services, specialist dementia support and FTD carer support spaces.

### Lack of general knowledge—barriers to support

However, despite carers being able to acknowledge key support that they found helpful, carers also identified significant barriers to accessing and receiving support necessary to help them in their roles as carers of a spouse with FTD. Twelve carers identified that the most significant barrier to receiving support was a lack of general knowledge about FTD, particularly amongst professional support services.

### Lack of appropriate services available

For 12 carers this lack of awareness about FTD meant that carers often felt that there were limited resources suitable to meet their specific needs.

*C14: And with the support group for dementia, [husband] will walk in and he'll go, "Yeah, I don't look anything like these people.* (Female, sporadic bvFTD)

Carers commonly described the younger age of their partner and the unique presentation of FTD behavioural symptoms as barriers to receiving appropriate support,

*C4: One of the problems I had when looking for respite for [husband] was that so many of the homes, you actually look and they all say, Oh yeah, we take people from the age of 65”[…] the thought of him at 55, being in a home where the next youngest person is 70.* (Female, sporadic bvFTD)

For carers, advice and support received from more general dementia support often did not seem appropriate to meet their specific carer needs. C6 describes how she felt different, and unable to relate to others when attending a dementia carer support course.

*C6: I'm doing a course through the Alzheimer's Society […] There's no one else young and there's no one else with FTD. And it is becoming apparent over the weeks how different my situation is to their situation.* (Female, fFTD)

### Limited accessibility and provision of services

Eight carers described having knowledge of an available service, but with barriers that interfered with their ability to access valuable services. Barriers included carers’ lack of knowledge about available resources and limited available resources in their area.

*C4: Yeah, the thing is, I find support quite confusing […] I get to the stage where I ask, “Well who are the people I see for this, and that?” […] I am aware that there is a reasonable amount of support in [my area], but as I say, it’s just knowing who to contact.* (Female, sporadic bvFTD)

Carers experienced a general lack of awareness about FTD amongst healthcare services, which may be linked to their experience of having limited availability of specialist support to meet the unique needs of spousal carers of people with bvFTD.

## Discussion

This is the first study to explore the experiences of spousal carers of people with bvFTD identifying support needs and barriers to meeting those needs. The findings highlight complex processes involved in receiving a diagnosis and adjusting to the role of spousal carer. This was impacted by the subtle onset of the condition, and further complicated by the challenging route to diagnosis and continued lack of support following diagnosis. Adjusting to the life altering, relationship changing and family impacts of becoming a carer to a person that is also a spouse was crucial in defining the spousal carer experience of bvFTD.

In line with previous literature [1] over half the carers experienced delays in diagnosis often because gradual behavioural symptoms were misinterpreted. The current study found that the lack of knowledge about FTD had a negative impact on carers. Information about common forms of dementia was insufficient due to the unique behavioural changes seen in bvFTD, which have a significant impact on spousal relationships. This lack of knowledge amongst healthcare professionals and the general public contributed to increased feelings of isolation, confusion, distress and frustration by carers. This meant that carers needed to play an active role in reaching diagnosis and advocating for the care of their spouse. Extending previous literature, carers described the complex process involved in receiving the bvFTD diagnosis, including experiencing mixed emotions, of relief, fear and a continued sense of isolation. There is a significant emotional impact of pre *and* post-diagnostic stages on carers which echoes findings from Chow and colleagues [31]. This is a time when carers are likely to be in greatest need of information on the symptoms of FTD [32].

Findings support existing literature emphasising that the receipt of diagnosis encompasses a difficult shift in the spousal relationship, which continues throughout the progression of FTD, and is characterised by a loss of communication, deep emotional connection, and companionship [21]. These changes are affected by behavioural symptoms of lack of empathy, apathy, loss of motivation and disinhibition, which often act to distance spouses or make communication and mutual understanding difficult [18], and therefore likely describe an experience that is specific to spousal carers of people with bvFTD. Carers likened this experience to having lost a partner that is still living with them. Previous qualitative studies describe how carers feel deep feelings of sadness and grief [20] as the spousal relationships becomes replaced by that of carer and care-recipient [19]. Furthermore participants described feelings of confusion and fear in response to the unpredictable nature of the behavioural changes, which at times included aggression, violence and high levels of risk, suggesting specific challenges and a greater emotional toll on carers of people with bvFTD, compared to other dementias. This is in line with prior studies describing the unique challenges of the FTD caring experience [31] and a greater burden [32] and distress [11] in caregivers of people with FTD compared to those with Alzheimer’s disease. Furthermore, behavioural changes have also been associated with caregiver depression in those caring for people with FTD [33].

Not previously reported in FTD carer literature this study explored the unique experiences of spousal carers of individuals with fFTD. This addressed the important implications of genetic disease on the family and included, issues surrounding testing and disclosure, parental feelings of helplessness regarding their ability to protect younger generations from developing FTD, and fearing for their family’s future. Although carers described mixed feelings about testing and disclosure, they acknowledged that knowing about the condition prior to diagnosis was helpful in preparing to manage expectations of their future together, and facilitated a process of accepting the diagnosis and adjusting to the carer role. A systematic review by Crook et al. (2021) found that while no studies specifically assessed the experience of genetic testing in fFTD, the wider FTD literature suggests that the carers and relatives of those with FTD often carry the responsibilities of providing consent on behalf of the patient and notifying family of the test results [33].

Carers developed ways of coping, some of which related to learning and implementing specific behavioural strategies, taking time for respite or shifting attitudes and perspectives towards their circumstances [14]. For example, participants described using strategies to manage challenging and risky behavioural symptoms, and supervising behaviour in a public, in line with reports from previous qualitative research [20]. Due to the progressive and changing presentation of the disease, carers’ coping must be adaptive. Therefore, carers’ responses indicate that the ability to cope fluctuates over time and this may be applied to the framework of the Lazarus and Folkman’s transactional model [34], which states that coping is not fixed and is dependent on an evolving relationship between an individual and their environment, as well as an individual’s appraisal of a stressor and their perceived ability to cope. As the disease progresses, caregiver’s appraisal of their ability to cope may decrease, resulting in an increased stress response.

The most important barrier reported by carers was the general lack of awareness about FTD, which impacts on diagnostic delays and support offered to carers. Participants expressed that they did not “fit in” amongst generic dementia support services, as they were insufficient in meeting the specific needs of spousal carers of people with bvFTD. Often due to younger age, familial and genetic implications and unique presentation of symptoms which dramatically impact on the spousal relationship and require specific behavioural and psychosocial coping skills on the part of the spousal carer. Therefore, findings emphasise a crucial need to educate healthcare professionals about FTD [22], and the initial symptoms and presentations, so that carers may be able to address concerns, receive timely diagnosis and access appropriate support [21].

### Limitations and future directions

The current study included more participants than other qualitative studies in the area of carer experiences of FTD [19, 20], and is an appropriate sample size for the TA. However, all participants were White British, residing in UK geographic areas comprising a majority of residents from upper income households and mostly female, which should be considered when addressing the transferability of the findings. Similarly, the sample also comprises of participants who are part of large cohort studies in a specialist dementia centre, therefore they are likely to be engaged with support and research teams and as such their experiences may not be generalisable to the wider carer experience. In addition, the reluctance of participants to personally identify as “carers” may hold implications that could provide information regarding crucial processes involved in transitioning into the carer role and the meaning of identifying as a carer or spousal carer. These were not explicitly taken into account during the process of the thematic analysis, which could have omitted this key aspect of the participants’ experience. Furthermore, while the researcher attempted to remove implicit biases, this heuristic frame of the “carer and patient”, as opposed to the “participant and spouse” may have biased the interpretations influencing the double hermeneutic process [35],whereby the researcher was attempting to make sense of the participant (as a carer) making sense of their experience (as a spouse and *not* a carer).

### Future research

The current study did not explore the impact on children living in the home during the early years of the disease, which may be of particular interest given that many carers report noticing symptoms up to twelve years before receiving a diagnosis, and acknowledge challenges in managing the responsibilities that come with a young family, alongside increasing responsibilities of caring. It will be important for research to further investigate the complex needs of young families with bvFTD, as well as explore the implications of the genetic condition on the family system.

### Clinical implications

Guidance by the National Institute of Health and Care Excellence [36] recommends that psychosocial support be provided for carers of people with dementia. However, the present study found that generalised dementia support offered to carers of people with FTD are insufficient to meet their needs. Therefore, it is important to emphasise public awareness campaigns and health professional education on bvFTD, as well as offering appropriate on-going support [21]. For example in the UK, educating general practitioners to improve the pre-diagnostic stage and journey to diagnosis and providing on-going support through memory services may be beneficial. Furthermore, families of people with fFTD may further benefit from receiving additional support regarding the genetic implications of the disease. Psychosocial interventions should be offered to support patients and families to cope with the specific challenges of bvFTD. Psychosocial interventions should include managing challenging behavioural symptoms, learning adaptive psychological coping strategies, engaging in FTD carer support groups as well as psychosocial support relating to grief, loss and processing the relationship change [11, 37–40].

## Conclusion

Healthcare professionals should be educated on the initial symptoms and presentations of FTD, so carers can address concerns, receive timely diagnosis and appropriate support. Families of people with FTD may benefit from receiving additional support in managing the genetic implications of the disease. Future research should investigate the needs of carers and families or people with fFTD, and would benefit from investigating positive or meaningful experiences in caring which may inform development of adaptive coping processes.

**Table 1:**
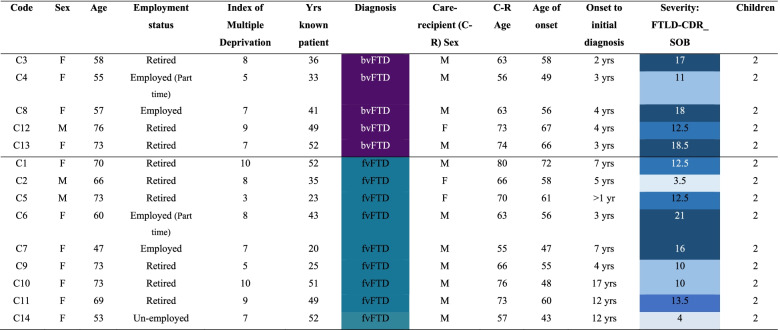
Participant Demographics

Key: FTLD-CDRSOB (O’Bryant et al., 2008): 4–7.5 (lowest severity); 8–11.5 (moderate severity); 12–15.5 (high severity); >16 (severe)

Multiple deprivation Indices (Dept. for Communities & Local Government, 2019): 1 is most deprived; 10 is least deprived

Diagnosis: fvFTD = bvFTD with familial; bvFTD = bvFTD only

**Table 2:**
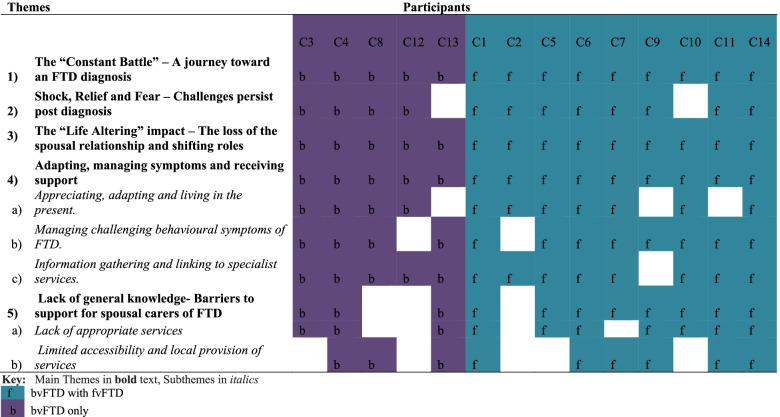
Representation of Themes Across Participants

## Supplementary Information

**Additional file1:** **AppendixA. **Interview Schedule

## Data Availability

The data analysing during the study are available from the corresponding author on reasonable request.
