# Supplementary material for: Exploring experiences and needs of spousal carers of people with behavioural variant frontotemporal dementia (bvFTD) including those with familial FTD (fFTD): a qualitative study
Source: BMC Geriatr. 2022 Mar 7;22:185. doi: 10.1186/s12877-022-02867-1 (PMC8900344; doi:10.1186/s12877-022-02867-1)
Supplement: Supplementary file 1 — Additional file1: AppendixA. Interview Schedule [file 12877_2022_2867_MOESM1_ESM.docx]

**Appendix A**

**Interview Schedule**

|  |  |  |
| --- | --- | --- |
|  | Interview Questions | *Examples of* *Optional prompts:* |
| 1 | I understand that you are a carer for a person with genetic/familial FTD (fFTD)/ behavioural subtype FTD (bvFTD). Could you tell me a bit about the person you care for and what your relationship is to them? |  |
| 2 | What was life like for you and the person you now care for, | *a. Can you tell me about when you noticed the initial symptoms and what that was like?* |
|  | before the FTD symptoms | *b. When were they diagnosed?* |
|  | emerged? | *c. What was it like to receive the diagnoses of fFTD/bvFTD and what does it mean?* |
|  |  | *d. Have you participated in genetic testing or FTD? Why or why not?*  *For fFTD carers:* |
|  |  | *a. What does it mean for you that the person you care for has a genetically-linked form of fFTD?* |
|  |  | *IF YES- What was this like for you?* |
| 3 | What does a typical day look like | a. *What has it been like for you?* |
|  | for you as a carer? | b. *What is your relationship like with the person you care for now?* |
|  |  | c. *What kinds of things do you think you experience as a carer for a person with bvFTD/fFTD that a carer of a person with general dementia wouldn’t?* |
| 4 | What kinds of things make caring more difficult? | a. *How do they make things for difficult for you?* |
| 5 | What kinds of things help you in your | a. *How do they help you?* |
|  | role as a carer? | b. *What kinds of things do you find particularly meaningful in your role as a carer for a person with bvFTD/fFTD?* |
|  |  | c. *What might it be like if you didn’t have these?* |
| 6 | How do you feel about the existing support you receive as a carer? | a. *Explore areas of Healthcare, social support, information* |
| 7 | What are the limitations of the existing support you have received so far? |  |
| 8 | What kinds of support do you think would help you in your role as a carer? | *a. How do you feel about Internet based support?* |
